# ICP-MS Method for Simultaneous Determination of Aluminum, Sodium, and Potassium in Human Albumin Solution for Infusion

**DOI:** 10.1155/ianc/2793979

**Published:** 2025-02-20

**Authors:** Sena Ozlem Gundogdu, Yeliz Aytimur, Seda Turhan, Adem Sahin

**Affiliations:** ^1^Department of R&D, Centurion Pharma, Ankara, Türkiye; ^2^Department of Pharmacy Service, Vocational School of Health Services, Bilecik Seyh Edebali University, Bilecik, Türkiye

**Keywords:** aluminum, human albumin solution for infusion, ICP-MS, potassium, sodium, validation

## Abstract

Elemental impurities in drug products may pose a risk to patient health. Therefore, maintaining the levels of these impurities below certain limits is essential for patient safety. Human albumin solution, one of the parenteral drugs used for many years, is crucial in various treatments. Also, the European Pharmacopoeia specifies limits for potassium, aluminum, and sodium in this drug. Inductively coupled plasma–atomic absorption spectrometry (ICP-AAS) and ICP–optical emission spectrometry (ICP-OES) are used for detecting elemental impurities. However, neither method can simultaneously analyze all three impurities within the pharmacopeial limits. This study aimed to develop a new method for simultaneously detecting the levels of potassium, aluminum, and sodium in human albumin–based drugs using ICP–mass spectrometry (ICP-MS). The limit of detection (LOD), specificity, linearity, repeatability, and accuracy were examined, and the recovery percentage was calculated. For Na, K, and Al elements, detection limits were calculated as 0.0105767 μg/mL, 0.001748 μg/mL, and 2.0568E − 4 μg/mL, respectively. Precision and reliability of this method have been proven by the linearity regression coefficients that were found as 0.999, 0.999, and 0.995 for Na, K, and Al. In addition, repeatability recovery rates were 98.70%, 98.38%, and 90.83%; accuracy analysis results were 101.45%, 94.53%, and 108.83% for 50% level; 98.26%, 93.93%, and 95.83% for 100% level; 100.48%, 95.90%, and 107.22% for 150% level for Na, K, and Al elements, respectively. This study successfully developed and validated ICP-MS for the simultaneous quantitative determination of the levels of potassium, aluminum, and sodium in human albumin solution.

## 1. Introduction

Human albumin is the most abundant macromolecular protein found in human plasma. It is synthesized by hepatocytes in the liver and released into the bloodstream [1]. Albumin mainly regulates the oncotic pressure exerted by plasma proteins and distributes internal body fluids across the body compartments [2]. Plasma albumin has a remarkable ligand-binding property and hence carries exogenous and endogenous compounds such as fatty acids, ligands, enzymes, and drugs. Thus, human serum albumin is used to cure patients suffering from hypovolemia, hypoalbuminemia, several liver diseases, resuscitation, blood loss, shock, and burns [3].

The production methods have improved over the years, and the analytical standards have been clearly defined in the pharmacopeia to reduce the adverse effects of drugs and provide patients with a more qualified and healthy life [4]. One of these standards is the determination of elemental impurities in drug products, which are crucial for human health. Therefore, keeping the levels of these impurities below certain limits is essential for patient safety, irrespective of whether these impurities are classified as excipients or contaminants.

The classifications and the factors responsible for the occurrence of the impurities must be considered while analyzing the drug products and setting the limits from the perspective of elemental impurities. The most common factors include the leaching of these impurities from the container closure systems, manufacturing processes, and natural occurrences. Based on the “Guideline for Elemental Impurities Q3D(R1)” in The International Council for Harmonization of Technical Requirements for Pharmaceuticals for Human Use (ICH), elemental impurities are classified as Class 1, Class 2, Class 3, and Other Elements. For Class 1, Class 2, and Class 3 impurities, control of the elemental impurities is operated by using the PDE values and toxicity of the related elements. These classes have their own requirements, and analyses are performed as per the ICH Q3D(R1) guideline. The toxicity and damage risk of other elements for human health are low, and hence these elements are controlled by other guidelines or approaches. Potassium, aluminum, and sodium are elemental impurities within the scope of this study, and they are included in the “Other Elements” group. Although they are considered less toxic elements, the qualitative and quantitative detection of these elements is crucial for human health [5–7].

According to the European Pharmacopoeia, the sodium and potassium levels are analyzed using atomic emission spectroscopy, and the aluminum levels are analyzed using atomic absorption spectroscopy. In previous studies, the levels of these three elements were examined mainly using inductively coupled plasma–atomic absorption spectrometry (ICP-AAS). The underlying principle is that the sample is sprayed into the flame with an oxidizing gas mixture and measured using light absorption by gas atoms and ICP–optical emission spectrometry (ICP-OES) that determines elemental concentrations using the light emitted from excited atoms and ions in the plasma. However, no methodology is available for analyzing the levels of these elements with ICP–mass spectrometry (ICP-MS), which provides precise measurements of elemental levels at extremely small concentrations based on the mass-to-charge ratio of the ion [8–12]. ICP-MS, ICP-OES, and ICP-AAS are multielement analytical techniques that can investigate trace elements both qualitatively and quantitatively. Their common advantages are their large analytical range, high sample throughput, low sample volume, and simple sample preparation. Among these three techniques, ICP-MS offers superior resolution and a low detection limit. In ICP-MS, the instrument contains two compartments: ICP and MS. In the ICP compartment, the liquid samples are transferred through the nebulizer to the carrier argon plasma, where the elevated temperature ionizes the analyte. The ionized analyte is carried to the mass spectrometer compartment through ion optics, where the sample is separated in the MS part based on its mass-to-charge (*m*/*z*) ratio. Then, the detector measures the ion concentration, generating the final data [13].

This study developed a new method using ICP-MS for the simultaneous determination of aluminum, sodium, and potassium in human albumin solution for infusion. The developed method was validated by calculating specificity, linearity, repeatability, and accuracy.

## 2. Materials and Methods

### 2.1. Materials

Nitric acid (65%) was procured from Merck. ICP-MS tuning solution (Beryllium (Be), 2 mg/L; indium (In), 0.4 mg/L; bismuth (Bi), 0.4 mg/L; cobalt (Co), 1 mg/L; manganese (Mn), 1 mg/L; cerium (Ce), 0.4 mg/L) was obtained from CPAchem. ICP-MS scandium was delivered as the internal standard at a concentration of 100 ppm (to the spray chamber using the internal standard peristaltic pump. Aluminum, sodium, and potassium standards were procured from AccuStandard. Ultrapure water was acquired from the Milli-Q system (Millipore, United States of America).

### 2.2. Methods

#### 2.2.1. Instrumentation

A Shimadzu ICP-MS-2030 device equipped with an AS-10 autosampler was used for the analyses. Also, 4% HNO_3_ solution was used as the washing solution, and the device contamination was controlled by applying this solution between sample injections. The operating conditions for the device are provided in Table 1.

#### 2.2.2. Method Development

**Figure 1:**
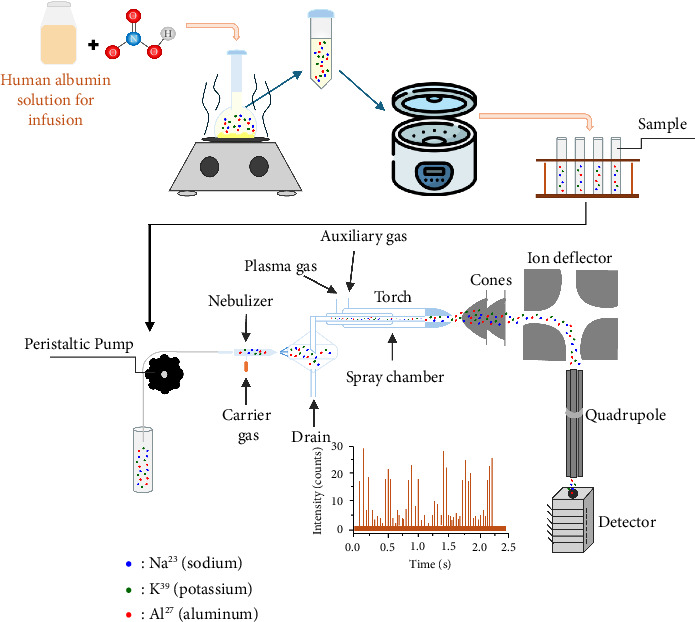
Na, K, and Al determination in human albumin solution by ICP-MS instrument.

Preparing samples with appropriate concentrations is crucial in obtaining more precise and accurate results by reducing device contamination for ICP-MS systems. Human albumin–based drugs contain significant amounts of protein macromolecules, and hence the probability of contamination must be considered delicately. To address this, we performed calculations and repeated sampling, optimizing the final concentration. The proteins were eliminated from the final analyte to detect elemental impurities in the sample solutions. A concentrated 65% of HNO_3_ was added, and heat was applied to start the xanthoproteic reaction that broke the peptide bonds in proteins. The temperature and time parameters were carefully controlled. The amount of nitric acid was adjusted considering device contamination and reaction initiation. Finally, the samples were centrifuged to remove the precipitate and obtain a clear solution for analysis. The duration of centrifugation and revolutions per minute (rpm) values were changed and set before starting the validation. The product was analyzed with each changed condition, and the recovery results were evaluated to obtain the most appropriate method. Finally, the optimized procedure for removal of the proteins from the experimental matrix was determined as the initiation of the reaction with the addition of 5 mL of 65% HNO_3_ to 0.5 mL of the product. After the initiation of the reaction, 125°C heat was applied for 2 h to accelerate the xanthoproteic reaction. At the end of the time, protein precipitation was observed in the volumetric flask and then removed. The remaining solution was carefully transferred to the centrifuge tube, and the solution was clarified by centrifugation at 6000 rpm for 20 min. The analyte at the end of the procedure has been used for the analysis of the elements. A schematic representation of the methodology is shown in Figure 1.

#### 2.2.3. Preparation of Solutions

##### 2.2.3.1. Calibration Solutions

For each element, the 100% level was considered as the target concentration, which was set as 3.90 μg/mL for potassium, 0.002 μg/mL for aluminum, and 23.0 μg/mL for sodium. Five separate solutions were prepared with target concentrations of 50%, 75%, 100%, 150%, and 200%. These concentrations were in the range of 1.95–7.80 μg/mL, 0.001–0.004 μg/mL, and 11.5–46.0 μg/mL for potassium, aluminum, and sodium, respectively. All the solutions were diluted with 2% HNO_3_ to a volume of 25 mL. Blank solution (2% HNO_3_) was used as the 0% reference point for the calibration curve.

The preparation of the calibration curves for each level is described below, each concentration level contains all three elements (Na, K, and Al):

• 50% ⟶ Take 287.5 μL Na, 487.5 μL K, and 25 μL Al and dilute to 25 mL with diluent. (C_Na_: 11.50 μg/mL, C_K_: 1.95 μg/mL, and C_Al_: 0.001 μg/mL)
• 75% ⟶ Take 431.5 μL Na, 731.5 μL K, and 37.5 μL Al and dilute it to 25 mL with diluent. (C_Na_: 17.25 μg/mL, C_K_: 2.93 μg/mL, and C_Al_: 0.0015 μg/mL)
• 100% ⟶ Take 575.0 μL μL Na, 975.0 μL K, and 50.0 μL Al and dilute it to 25 mL with diluent. (C_Na_: 23.00 μg/mL, C_K_: 3.90 μg/mL, and C_Al_: 0.002 μg/mL)
• 150% ⟶ Take 862.5 μL Na, 1462.5 μL K, and 75.0 μL Al and dilute it to 25 mL with diluent. (C_Na_: 34.50 μg/mL, C_K_: 5.85 μg/mL, and C_Al_: 0.003 μg/mL)
• 200% ⟶ Take 1150.0 μL Na, 1950.0 μL K, and 100.0 μL Al μL and dilute it to 25 mL with diluent. (C_Na_: 46.00 μg/mL, C_K_: 7.80 μg/mL, and C_Al_: 0.004 μg/mL)

##### 2.2.3.2. Unspiked Sample Solution

For this, 0.5 mL of the finished product (albumin 20%, 100 mL solution for infusion) was transferred to a 50-mL volumetric flask. Then, 5 mL of 65% HNO_3_ was added and shaken slightly. After dissolution, the solution was diluted with pure water and heated at 125°C for 2 h. The heated sample was transferred into a centrifuge tube and centrifuged at 6000 rpm for 20 min. The supernatant was carefully transferred into the sample container without shaking the tube and used for analysis.

##### 2.2.3.3. Spiked Sample Solution

For this, 0.5 mL of the finished product (albumin 20%, 100 mL solution for infusion) was transferred to a 50-mL volumetric flask. 1150.0 μL Na, 1950.0 μL K, and 100.0 μL Al were added onto the sample. Then, 5 mL of 65% HNO_3_ was added to the flask and shaken slightly. After dissolution, the solution was diluted with pure water and heated at 125°C for 2 h. The heated sample was transferred into a centrifuge tube and centrifuged at 6000 rpm for 20 min. The supernatant was carefully transferred into the sample container without shaking the tube and taken for analysis. (C_Na_: 23.00 μg/mL, C_K_: 3.90 μg/mL, and C_Al_: 0.002 μg/mL).

#### 2.2.4. Validation

The specificity, linearity, accuracy, and precision were determined using ICP-MS to determine the levels of potassium, aluminum, and sodium in human albumin solutions, except for the 24 elements specified in Q3D (R1), which were studied in accordance with the validation parameters and limits specified in USP < 233 > guideline. Validation parameters and limits are clearly specified in USP < 233 >, and this study was performed in accordance with the defined procedure taking into account the acceptance criteria, and the validation was performed as per the ICH Q2 (R2) guideline [14, 15]. Before starting the analysis, system suitability was assessed to confirm whether the system was working appropriately and provide precise and accurate results. The blank solution, calibration standards, spiked sample, and unspiked sample were prepared to evaluate specificity. The selectivity of each element was evaluated by comparing the interaction of that specific element in the blank and sample solutions. The linearity was examined by preparing calibration samples from the standard solution at concentrations of 0%, 50%, 75%, 100%, 150%, and 200%, and the analyses were performed from each calibration level with three replicates. The accuracy was determined by using spiked sample solutions at concentrations of 50%, 100%, and 150%, with three replicates per level, resulting in nine spiked samples. Recovery percentages of the elements were calculated to assess the method compatibility. Precision was examined to validate the proximity of replications derived from the same sample. Three parameters, including system precision, repeatability, and intermediate precision, were conducted and combined to obtain overall precision. System precision was applied to determine the consistency of repeatability in the standard solution analysis. The RSD% value between six injections of the 100% solution was calculated in the linearity study. The repeatability was applied to determine the consistency of the six different 100% spiked sample solutions to observe the RSD% values. The repeatability analysis was reperformed on a different day with a different analyst for intermediate precision. The results of this analysis were combined with the repeatability analysis results (total number of analytes 12) and evaluated. In addition, the limit of quantification (LOQ) and limit of detection (LOD) values were calculated to determine the lowest concentrations for each element.

## 3. Results and Discussion

Potassium, aluminum, and sodium were analyzed in the human albumin solution for infusion, referring to the European Pharmacopoeia, 11th Edition. Sodium caprylate and sodium chloride were added to the product formulation while preparing the albumin product, and sodium hydroxide was used for pH adjustment [11]. The analyzed product within the scope of this study consisted of sodium caprylate as the stabilizer and sodium chloride for adjusting osmolality. The final concentration of sodium was 23.00 μg/mL. As the European Pharmacopoeia sets the sodium limit as 95%–105%, the sodium concentration was kept between 21.85 and 24.15 μg/mL. If the sodium concentration in the albumin solution exceeds the limits, it may increase blood pressure and cause hypervolemia, resulting in heart, liver, and/or kidney failure [16]. On the contrary, the potassium limit has been determined as the maximum 3.90 μg/mL of potassium per gram of protein. Potassium is the most abundant electrolyte found in the intracellular fluid. It maintains the intracellular and extracellular osmotic balance in the human body. Elevating the potassium concentration in the body may disrupt this balance, resulting in decreased blood pressure and reduced intravascular fluid. This is defined as hyperkalemia and may cause cardiac arrhythmias, muscle weakness, or paralysis [17]. Finally, the limit for aluminum is determined as the maximum 2.0 × 10^2^ μg/L. The main reason for analyzing aluminum as an elemental impurity in human albumin solution is the high probability of glass-borne contamination. The high dose of aluminum may be toxic to the brain, respiratory system, parathyroid gland, kidneys, liver, bones, and bone marrow [18]. The potential occurrence of any of these effects can substantially decrease the patient's quality of life or even result in mortality. Although ICH has not set any limits for these three elements, analyzing and detecting them is important for the aforementioned reasons. Thus, the European Pharmacopoeia sets limits for these elements specific to the human albumin solution.

**Figure 2:**
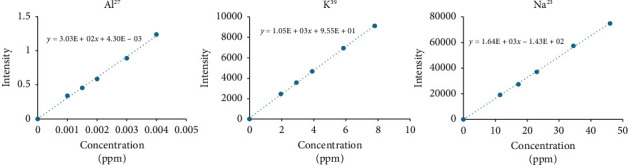
Linearity graphs for aluminum (Al^27^), potassium (K^39^), and sodium (Na^23^) elements, respectively.

The solutions with target concentrations of 0%, 50%, 75%, 100%, 150%, and 200% were prepared, and the calibration curves were obtained for sodium, aluminum, and potassium with three replicate readings to calibrate the system. In calibration curves, the correlation coefficients for all elements were found to be higher than 0.99. The linearity graphs plotted with the calibration data are shown in Figure 2, and linearity, LOQ, and LOD values for each element are listed in Table 2. The LOQ values were found as 0.019222 μg/mL for sodium, 0.005827 μg/mL for potassium, and 6.8559E − 4 μg/mL for aluminum. The detected LOQ values for each element were extremely low, thereby proving the sensitivity of the developed method. Elements below the LOQ values do not pose any threat and therefore require no evaluation in terms of toxicity. The results of the product analysis (Table 3) showed that the analysis results for the three elements were below the LOQ values, and they posed no risk to the patient's health during drug use.

The consistency of the study was validated by analyzing six repeatability sample solutions spiked with 100% of the target concentrations for each element. The upper RSD% limits for sodium and potassium were 10%, and for aluminum, the limit was 20%. The results obtained were below the predicted limits. The intermediate precision was analyzed using different analysts at different times, and the results of the six spiked sample solutions were combined with the results of the repeatability study. The data obtained from a combination of 12 spiked samples were evaluated. The RSD% limits of the corresponding study were determined as 15% for sodium and potassium and 25% for aluminum. The limits and results are shown in Table 4.

For evaluating accuracy, spiked samples were prepared in triplicate at 50%, 100%, and 150% of each target concentration, with a total of nine injections performed. For these injections, the recovery percentage was calculated as 95.0%–105.0% for sodium and 90%–110% for potassium and aluminum. The results of the study are displayed in Table 5.

When the analysis methods specified in the pharmacopoeia are compared with existing analysis methods, it can be seen that Jarošová et al. compared the toxic elements in coffee by analyzing them with AAS and ICP-MS. In this study, it was stated that the ICP-MS analysis was faster than the AAS method, and when the running cost of the methods were examined, it was emphasized that the cost of the analysis with AAS may be slightly higher. Although the initial investment cost is high, the advantage of the simultaneous analysis by ICP-MS has been demonstrated [19]. Another comparison was made in the technical note prepared by the Horiba Group. In this technical note, it is seen that ICP-MS can analyze the aluminum level much more sensitively compared to flame AAS and graphite furnace AAS methods. On the other hand, the need for trained personnel, high investment costs, and higher operation costs are seen as disadvantages of the ICP-MS method in comparison [20]. In today's conditions, where the analysis of elemental impurities has become a necessity, where ICP-MS investment has already been made and trained personnel are available in many laboratories, this method, which allows simultaneous analysis of potassium, aluminum, and sodium in the albumin solution product, can be considered as a suitable alternative.

After completing the validation study and meeting the determined acceptance criteria for each parameter, drug product analyses were performed. Two different batches of 20% albumin solution for infusion were analyzed quantitatively to determine the levels of sodium, potassium, and aluminum in the drug product. The results of one batch are shown in Table 3 as a representative example.

## 4. Conclusions

Human albumin solution for 20% infusion is used for treating patients with human albumin deficiency. The levels of sodium, potassium, and aluminum in this drug are restricted by the European Pharmacopoeia monograph. The monograph includes analytical procedures for determining the levels of these elements using ICP-AES and ICP-OES. This study was novel in developing and successfully validating ICP-MS for the simultaneous quantitative determination of the levels of sodium, potassium, and aluminum in human albumin solutions. The validation results met the requirements for each parameter examined in this study. Hence, this study demonstrated that using the developed ICP-MS method allows for accurate determination of sodium, potassium, and aluminum levels in human albumin solution for 20% infusion.

## Figures and Tables

**Table 1:** ICP-MS operating conditions.

Conditions	Properties
RF power	1.20 kW
Sampling depth	5.0 mm
Plasma gas	Ar: 9.0 L/min
Auxiliary gas	Ar: 1.10 L/min
Carrier gas	Ar: 0.70 L/min
Torch	Mini-torch ICPMS
Nebulizer	Nebulizer O7UES
Chamber	Cyclone chamber
Chamber temperature	5°C
Number of scan	20
Cell gas	He: 6.0 mL/min
Cell voltage	−21 V
Energy filter	7 V
Solvent rinse time	10 s (low) 30 s (high)
Sample rinse time	30 s (low) 30 s (high)
Peristaltic pump rotation speed	20 rpm (low) 60 rpm (high)

**Table 2:** Linearity results and LOD-LOQ values of Na, K, and Al elements.

Elements	Slope	Intercept	Linear range (ppm)	*R*	LOD (ppm)	LOQ (ppm)
Na	1.64E + 03	−1.43E + 02	11.5–46.0	0.999	0.005767	0.019222
K	1.05E + 03	9.55E + 01	1.95–7.80	0.999	0.001748	0.005827
Al	3.03E + 02	4.30E − 03	0.001–0.004	0.995	2.0568E − 4	6.8559E − 4

**Table 3:** Finished product (albumin 20%, 100 mL solution for infusion) analysis results.

Elements	Ph. eur. limits (ppm)	Results (ppm)
Na	21.85–24.15	22.90
K	≤ 3.90	0.00
Al	≤ 0.0020	0.0004

**Table 4:** Repeatability and intermediate precision results.

Elements	Repeatability (*n* = 6)		Intermediate precision (*n* = 6)		RSD% (*n* = 12)
	Average results (ppm)	RSD %	Average results (ppm)	RSD %	
Na	45.60	1.51	45.88	1.27	1.37
K	3.84	1.26	3.99	0.98	2.29
Al	0.0022	4.44	0.0021	7.28	6.74

**Table 5:** Accuracy results.

Elements	Average recovery for 50% level	Average recovery for 100% level	Average recovery for 150% level	Total recovery
Na	11.67 ppm (101.5%)	22.60 ppm (98.3%)	34.67 ppm (100.5%)	22.98 ppm (100.1%)
K	1.84 ppm (94.5%)	3.66 ppm (93.9%)	5.61 ppm (95.9%)	3.70 ppm (94.8%)
Al	0.0011 ppm (108.3%)	0.0020 ppm (95.8%)	0.0030 ppm (107.2%)	0.0019 ppm (103.8%)

## Data Availability

The data that support the findings of this study are available from the corresponding author upon reasonable request.
